# The XRCC1 phosphate-binding pocket binds poly (ADP-ribose) and is required for XRCC1 function

**DOI:** 10.1093/nar/gkv623

**Published:** 2015-06-29

**Authors:** Claire Breslin, Peter Hornyak, Andrew Ridley, Stuart L. Rulten, Hana Hanzlikova, Antony W. Oliver, Keith W. Caldecott

**Affiliations:** Genome Damage and Stability Centre, School of Life Sciences, University of Sussex, Falmer, Brighton BN1 9RQ, UK

## Abstract

Poly (ADP-ribose) is synthesized at DNA single-strand breaks and can promote the recruitment of the scaffold protein, XRCC1. However, the mechanism and importance of this process has been challenged. To address this issue, we have characterized the mechanism of poly (ADP-ribose) binding by XRCC1 and examined its importance for XRCC1 function. We show that the phosphate-binding pocket in the central BRCT1 domain of XRCC1 is required for selective binding to poly (ADP-ribose) at low levels of ADP-ribosylation, and promotes interaction with cellular PARP1. We also show that the phosphate-binding pocket is required for EGFP-XRCC1 accumulation at DNA damage induced by UVA laser, H_2_O_2_, and at sites of sub-nuclear PCNA foci, suggesting that poly (ADP-ribose) promotes XRCC1 recruitment both at single-strand breaks globally across the genome and at sites of DNA replication stress. Finally, we show that the phosphate-binding pocket is required following DNA damage for XRCC1-dependent acceleration of DNA single-strand break repair, DNA base excision repair, and cell survival. These data support the hypothesis that poly (ADP-ribose) synthesis promotes XRCC1 recruitment at DNA damage sites and is important for XRCC1 function.

Single-strand breaks (SSBs) are the commonest lesions arising in cells, resulting both directly from disintegration of deoxyribose and indirectly during the excision repair of DNA base damage [reviewed in (1)]. SSBs usually lack conventional 3′-hydroxyl and 5′-phosphate termini, often possessing modifications such as 3′-phosphate or 5′-hydroxyl termini, or fragments of deoxyribose or topoisomerase. If not repaired rapidly, such termini can block progression of RNA or DNA polymerases, disrupting transcription or replication, respectively. The threat posed by SSBs is indicated by the existence of human genetic diseases associated with neurological dysfunction in which single-strand break repair (SSBR) is attenuated (1).

To date, all known SSBR-defective diseases are associated with defects in end processing, the step of repair during which conventional 3′-hydroxyl and 5′-phosphate termini are restored. One critical component of end processing is XRCC1, a molecular scaffold protein that interacts with and recruits, stabilizes, and/or stimulates end processing enzymes and accelerates SSBR ∼5-fold (2,3). The importance of XRCC1 is illustrated by the hypersensitivity of XRCC1-mutant cells to a broad range of genotoxins and to their elevated frequency of chromosome aberrations, genetic deletions and sister chromatid exchanges (4–6). Moreover, mice with conditional deletion of *Xrcc1* in brain recapitulate many of the pathologies associated with loss of SSBR in humans, including cerebellar defects, ataxia and seizures (7).

A number of observations suggest that XRCC1 recruitment at chromosomal SSBs is promoted by poly (ADP-ribose) (PAR) synthesis. First, XRCC1 interacts directly with both PAR and with the poly (ADP-ribose) polymerases PARP1 and PARP2 (8–10). Second, small molecule-mediated inhibition of PAR synthesis, or depletion/deletion of PARP1, greatly reduces the accumulation of XRCC1 at sites of H_2_O_2_ or UV laser-induced DNA damage (11–15). Third, mutations that disrupt folding of the PAR-binding BRCT1 domain in XRCC1 reduce or ablate XRCC1 accumulation at DNA damage (9,11,15,16). Finally, depletion of PARG, the enzyme responsible for PAR degradation following SSBR, increases both steady state cellular levels of PAR and the accumulation and/or persistence of XRCC1 in sub-nuclear foci before and after DNA damage (17).

Despite these observations, however, several recent reports have challenged the importance of PAR binding for XRCC1 function, instead ascribing XRCC1 recruitment to DNA binding protein partners such as DNA polymerase β (Polβ), polynucleotide kinase/phosphatase (PNKP), and DNA ligase IIIα (Lig3α) (18–22). One reason this uncertainty remains is a lack of clarity concerning the mechanism of PAR binding by XRCC1. PAR binding was first ascribed to a degenerate motif present at the C-terminus of the central BRCT1 domain in XRCC1, comprised of an alternating series of basic/hydrophobic residues and present in numerous other PAR binding proteins (9). However, a recent study instead assigned PAR binding to the phosphate binding pocket present in the BRCT1 domain (16). Not knowing the site of PAR interaction has prevented the generation of point mutations that specifically reduce or ablate PAR binding, and consequently an analysis for their impact on XRCC1 function. Here, we have confirmed the site of PAR binding in XRCC1, enabling us to mutate this site and address directly, for the first time, its importance for XRCC1 cellular function.

## MATERIALS AND METHODS

### Cell lines

The osteosarcoma cell line U2OS (obtained from the Genome Damage and Stability Centre cell repository) and derivatives of the Chinese hamster ovary (CHO) cell line EM9 (4) were maintained as monolayers in modified Eagle's medium (MEM) or Dulbecco's modified Eagle's medium (DMEM), respectively, supplemented with 10% (vol/vol) foetal calf serum, 100 U/ml penicillin, 2 mM glutamine and 100 μg/ml streptomycin. Expression constructs were introduced into the *XRCC1*-mutant CHO cell line EM9 by Genejuice transfection (Novagen) and stable cell lines prepared by selection in media containing 1.5 mg/ml G418 (Gibco-Invitrogen) for 10–14 days. The cell line U2OS^GFP-XRCC1^ was generated by transfection of 1 × 10^6^ U2OS cells with 0.5 μg pEGFP-XRCC1 by nucleofection (Lonza kit V) according to the manufacturer's instructions. Twenty four hours after nucleofection, cells were selected in media containing 1 mg/ml G418 for 3 weeks and single clones selected based on their level of GFP expression. One clone, denoted U2OS^GFP-XRCC1^, was selected for further use.

### XRCC1, PARP1 and PCNA expression constructs

To create pCD2EXH^R335A,K369A^ (denoted pcD2EXH^RK^), encoding human XRCC1-His ^R335A,K369A^, the *XRCC1* ORF in pCD2EXH (23) was mutated using a QuikChange Mutagenesis Kit (Agilent Technologies) using the oligonucleotides 5′-CCAGAACCCCTTCGCCTCCGAGCTGCGAG-3′ and 5′-TGCCAACACCCCCGCGTACAGCCAGGTCC-3′. All mutated ORFs were confirmed by sequencing. To create pmRFP-XRCC1^161–406^ and pmRFP-XRCC1^161–406 RK^, encoding human mRFP-XRCC1^161–406^ and mRFP-XRCC1^161–406 RK^, respectively, cDNA encoding XRCC1 residues 161–406 was amplified by PCR using the oligonucleotides 5′- AAGAATTCCATGCACCATCACCATCACCATCCGTCCCAGAAGGTGACAGTG-3′ (forward) and 5′- CCCGAATTCTGCAGTCATGGCCCTGCCATGAGGTA-3′ (reverse) and either pCD2EXH or pCD2E-XH^RK^ as template, as appropriate. Note that these constructs also possess an N-terminal histidine tag (underlined). PCR products were verified by sequencing and cloned into the *Eco*R1 site of pmRFP (24). PARP1-pmCherry and PARP1^E988K^-pmCherry were kind gifts from Gyula Timinszky (Ludwig-Maximilians University, Munich). pCCC-TagRFP is a chromobody-Tag plasmid encoding PCNA-V_H_H fused to RFP (Chromotek), which enables detection of endogenous PCNA.

### Western blotting

Proteins were fractionated on 8% or gradient SDS-PAGE gels and transferred onto nitrocellulose membrane (GE Healthcare). Membranes were blocked in 5% non-fat dried milk/TBST for 30 min at room temperature or overnight at 4°C. After washing, blocked membranes were incubated with rabbit anti-GFP polyclonal antibody (#2555, Cell Signalling Technology), rabbit anti-phospho (pS485/pT488) XRCC1 polyclonal antibody (1:5000; A300-231A; Bethyl Laboratories, Inc.), mouse anti-PAR (10H) Mab (1:1000), mouse anti-PARP1 Mab (1:1000, MCA1522G, Bio-Rad), mouse anti-Actin Mab (1:2000, A4700, Sigma), mouse anti-XRCC1 (33-2-5) Mab (23), or mouse anti-polyhistidine (His-tag) Mab (Sigma). Anti-mouse and anti-rabbit HRP-conjugated secondary antibodies (Dako Cytomation) were employed at 1:5000, in 5% non-fat milk for 1 h at room temperature. Detection was by ECL (GE Healthcare) and autoradiography.

### Transfection and fluorescence imaging

U2OS cells or U2OS^GFP-XRCC1^ cells were seeded onto coverslips and transfected 1 day later with appropriate constructs using FuGENE 6 transfection reagent according to the manufacturer (Promega). Twenty four hours later, the cells were mock-treated or treated with 10 mM H_2_O_2_ for 10 min, incubated at 37°C in drug free media for 15 min, washed with phosphate buffered saline (PBS) and then fixed for 10 min in 4% paraformaldehyde in PBS at room temperature. After fixation the cells were washed 2× with PBS, treated with ice-cold methanol/acetone solution for 10 min, washed 2× with PBS and mounted using VECTASHIELD Mounting Media. Images were captured on a Leica SP8 confocal microscope. For EdU labeling of sites of DNA replication, the Click-iT^®^ EdU Alexa Fluor^®^ 647 Imaging Kit from Molecular Probes was used according to manufacturer's instructions. For laser microirradiation, 2 × 10^5^ EM9 cells were seeded onto glass-bottom dishes (Mattek) and transfected with 1 μg of the indicated pmRFP-XRCC1 construct using Genejuice (Milipore). Twenty four hours later, cells were pre-incubated with 10 μg/ml Hoechst 33258 (for micro-irradiation with a 351 nm laser) or Hoechst 34580 (for a 405 nm laser) for 30 min prior to localised micro-irradiation with a 351 nm or 405 nm UV-laser at a dose of 0.22 J/m^2^ as previously described (25). Time-lapse images were recorded at the intervals shown after micro-irradiation. For experiments with PARP inhibitor, cells were pre-incubated with either 100 nM Olaparib (Selleckchem) as indicated or with 500 nM Ku58948 (AstraZeneca) 30 min before micro-irradiation.

### Clonogenic survival assays

The indicated cells (500) were plated in duplicate in 10 cm dishes and incubated for 4 h at 37°C. Cells were rinsed with PBS and either mock treated or treated with H_2_O_2_ (diluted in PBS at the indicated concentration immediately prior to use) or methyl methanosulfonate (MMS) (diluted in complete medium at the indicated concentration immediately prior to use) for 15 min at room temperature (H_2_O_2_) or 37°C (MMS). After treatment, cells were washed twice with PBS and incubated for 10–14 days in drug-free medium at 37°C to allow formation of macroscopic colonies. Colonies were fixed in ethanol (95%), stained with 1% methylene blue in 70% ethanol and colonies of >50 cells counted. Percentage survival was calculated for each drug concentration using the equation 100 × [average mean colony number (treated plate)/average mean colony number (untreated plate)].

### Alkaline single cell agarose gel electrophoresis (alkaline comet assay)

Sub-confluent cell monolayers were trypsinised, diluted to 2 × 10^5^ cells/ml in ice-cold PBS (for H_2_O_2_ treatment) or complete media (for MMS treatment) immediately prior to treatment, and either mock-treated or treated with 150 μM H_2_O_2_ (diluted in ice-cold PBS immediately prior to use) for 20 min on ice or with the indicated concentration of MMS (diluted in complete medium) for 15 min at 37°C. Cells were then rinsed in ice-cold PBS and incubated, where appropriate, in fresh drug-free media for the desired repair period at 37°C. Cells (100 per data point) were then analysed by alkaline comet assay as previously described (26) using Comet Assay IV software (Perceptive Instruments).

### Expression and purification of His-XRCC1^161–406^ and His-XRCC1^161–406 RK^

For expression of recombinant XRCC1 proteins, we employed Rosetta^TM^2 (DE3)pLysS (Merck Millipore) *Escherichia coli* harbouring the expression plasmids pTWO-E-His-XRCC1^161–406^ or pTWO-E-His-XRCC1^161–406 RK^. pTWO-E is modified from pET-17b, encoding an N-terminal Rhinovirus 3C-cleavable, His_6_ affinity tag. For XRCC1 expression, 100 ml LB ampicillin/chloramphenicol media was inoculated with a single bacterial colony and incubated with shaking (220 rpm) at 37°C for 8 h and then stored at 4°C overnight. The next day, 6× 1 l of LB-ampicillin/chloramphenicol media supplemented with antibiotics as above was inoculated with the starter culture (10 ml/l) and again incubated, with shaking, at 37°C until and OD_600_ of 0.8–1.0 was reached, after which protein expression was induced by the addition of 0.2 ml 1 M IPTG/litre for a period of 3 h at 30°C. Cells were harvested by centrifugation and the resulting pellet stored at −20°C. For purification, cell pellets were thawed on ice, resuspended in 50 mM HEPES pH 7.5, 250 mM NaCl, 10 mM imidazole and 1 mM Tris(2-carboxyethyl)phosphine (TCEP), and lysed by sonication on ice for 10 min (10 s on/10 s off) using a large parallel probe at 25% amplitude (Sonics Vibra-Cell, VWR). The lysate was clarified by centrifugation for 50 min at 40 000 × *g* at 4°C and the resulting supernatant added to a 5 ml bed volume of Talon resin (Clontech) in a gravity flow column. After 30 min incubation with the resin, with mixing at 4°C, unbound material was removed by sequential washes (3 × 10 ml) with resuspension buffer. Bound protein was eluted by addition of (2 × 5 ml) elution buffer (50 mM HEPES pH7.5, 250 mM NaCl, 300 mM imidazole and 1 mM TCEP). The eluate was loaded onto a pre-equilibrated (20 mM HEPES pH 7.5, 150 mM NaCl and 1 mM TCEP) 5 ml FF Heparin column (GE Healthcare) and bound material eluted with a linear salt gradient (20 mM HEPES pH 7.5, 1 M NaCl and 1 mM TCEP). Fractions containing XRCC1 were identified by SDS-PAGE, then pooled and concentrated, using Vivaspin 20 (10 000 MWCO) centrifugal concentrators (Sartorius Stedim), to a final concentration of 0.3 mg/ml, and then stored at −80°C until required.

### Thermal denaturation and circular dichroism

For thermal denaturation, samples containing 2.0 μM protein and 5× SYPRO Orange (diluted from a 5000 × stock supplied in DMSO; catalogue number S5692, Sigma–Aldrich) were prepared in sample buffer [50 mM HEPES pH 7.5, 300 mM NaCl, 0.5 mM TCEP and 5× Sypro Orange (from Sigma–Aldrich^®^)]. Denaturation curves were monitored in 96-well PCR plates in a Roche LightCycler 480 II, using 465 and 580 nm filters for excitation and emission wavelengths, respectively. Temperature midpoints (*T_m_*) for each folded to unfolded transition were determined by non-linear regression fitting of a modified Boltzmann model (27) to normalized data in Prism5 (GraphPad Software).

\begin{equation*} Y = (a_n X + b_n ) + \frac{{(a_d X + b_d ) - (a_n X + b_n )}}{{1 + e^{\frac{{T_m - X}}{m}} }} \end{equation*}

where: *a_n_* and *a_d_* are the slopes, *b_n_* and *b_d_* the *y*-intercepts, of the native and denatured baselines, respectively. *T_m_* is the melting temperature and *m* a slope factor.

For circular dichroism, spectra were measured at 20°C between the wavelengths 198 and 280 nm in a JASCO J-715 spectropolarimeter attached to a JASCO PTC-384W temperature control system. CD spectra were measured using a 0.1 mm path length cell (Starna Scientific), with protein at a concentration of 54 μM, that had been buffer-exchanged into 10 mM HEPES pH 7.5, 300 mM NaCl, 0.5 mM TCEP. Spectra were measured using a 0.1 mm path length cell (Starna Scientific) and represent the average of 10 consecutive scans, where the signal from buffer alone has been subtracted.

### Poly (ADP-ribose) binding assays

The wells of flat bottomed 96 well PS-microplates (Greiner) were incubated with either 50 μl recombinant histone H1, PARP1 or BSA at 0.1 mg/ml in PBS overnight at 4°C and the wells rinsed (4×) with 0.2 ml 0.1% Triton X100 in PBS. The adsorbed proteins were mock ribosylated in the absence of NAD^+^ or ribosylated in the presence of the indicated concentration of NAD^+^ (Sigma) in PARP1 reaction buffer (50 mM Tris–HCl pH8, 0.8 mM MgCl_2_, 1% glycerol and 1.5 mM DTT) containing 40 nM single-stranded oligodeoxyribonucleotide (Eurogentec: 5′-CATATGCCGGAGATCCGCCTCC-3′) and 5 nM PARP1 (recombinant, human, full length) in a final volume of 50 μl at room temp for 30 min. After rinsing (4×) with 50 μl of 0.1% Tween 20 in PBS, 50 μl of His-XRCC1^161–406^ or His-XRCC1^161–406 RK^ (diluted to 25 nM in 20 mM Tris pH7.5, 130 nM NaCl) was added to the adsorbed proteins and incubated on ice for 30 min. Where indicated, His-XRCC1 proteins were pre-incubated with mono (ADP-ribose) or poly (ADP-ribose)(Trevigen) competitor at the concentrations indicated for 30 min at 4°C, before their addition to the adsorbed proteins. The wells were then rinsed (4×) as above and incubated with 50 μl mouse anti-polyhistidine (His-tag) Mab (Sigma, diluted 1: 3000 in 20 mM Tris pH7.5, 130 nM NaCl) followed by 50 μl HRP-conjugated rabbit anti-mouse IgG (Dako, 1: 5000 in dilution buffer) for 30 min each on ice. After a final wash with 0.1% Tween 20 in PBS, 50 μl of TACS Sapphire (Trevigen) was added to the wells, incubated in the dark for 15 min, stopped by adding 0.2 M HCl, and the absorbance was read at 450 nm.

### GFP pull down experiments

U2OS^GFP-XRCC1-His^ cells (see above), or U2OS cells 48 h after nucleofection (Amaxa; Lonza, Slough, UK) with 4 μg each of pEGFP-XRCC1^161–406^ or pEGFP-XRCC1^161–406 RK^ and either pmCherry-PARP1 or pmCherry-PARP1*^E988K^* were snap frozen until needed. Cells were then thawed on ice and lysed on ice for 20 min in 0.4 ml/5 × 10^6^ cells in 25 mM HEPES (pH 7.8), 150 mM NaCl, 10% glycerol, 0.5% Triton X-100, and including Protease Inhibitor Cocktail and Phosphatase Inhibitor Cocktail 3 (Sigma-Aldrich^®^, Dorset, UK). Where indicated, the PARP1 inhibitor KU58948 (500 nM) was added to the cell culture medium 1 h prior to cell harvest and/or was included in the cell lysis buffer. Lysed cells were sonicated in a Bioruptor and clarified by centrifugation at 4°C. Unless stated otherwise, all subsequent steps were performed on ice. Forty microliters of the clarified extract was retained on ice as ‘input’ and 360 μl was mixed with 15 μl (bed volume) of GFP-Trap_A beads (ChromoTek GmbH, Germany) prewashed in 0.5 ml wash buffer (lysis buffer containing 1 mM DDT and 25 mM imidazole). After 1 h on a carousel at 4°C, the GFP-Trap^®^_A beads were gently pelleted by centrifugation at 2000 × *g* for 2 min. Sixty microliters of the supernatant was retained as ‘unbound’ material and the pellet was washed three times in wash buffer, with 50 μl of the final wash retained as ‘final wash’. Proteins were eluted from the beads by re-suspension in 50 μl 2× Laemmli buffer (250 mM Tris (pH 8.0), 10% SDS, 500 mM DTT, 50% glycerol)), heating for 5 min at 95°C, and centrifugation at 2700 × *g* for 2 min to recover the supernatant.

## RESULTS

**Figure 1.**
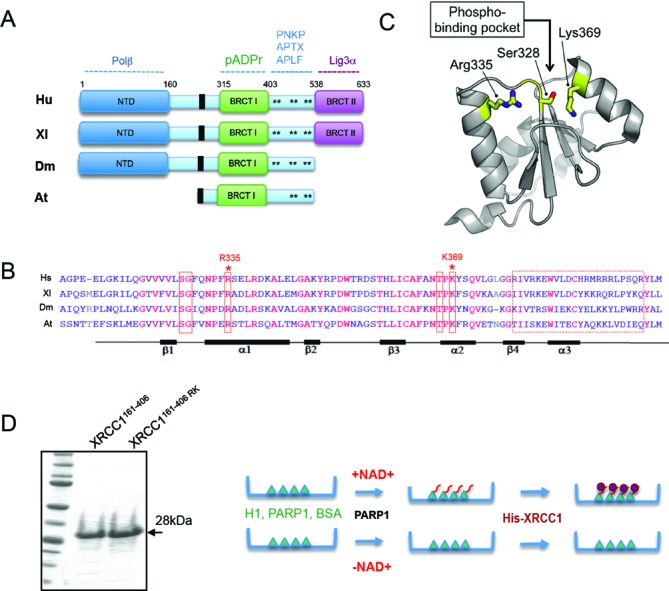
Conservation of the XRCC1 BRCT1 domain and its phosphate-binding pocket. (**A**) Schematic depicting conservation of XRCC1 domains in human (Hs), frog (Xl), fly (Dm), and plant (At) XRCC1. Binding sites for the indicated protein partners are shown. Asterisks denote CK2 phosphorylation sites that mediate FHA-dependent interactions with PNKP, APTX and APLF. Black boxes denoted the nuclear localization signal. (**B**) Alignment of the BRCT1 domain from human, frog, fly and plant. Solid red boxes denote the residues predicted to form the phosphate-binding pocket and the dotted red box denotes the degenerate putative PAR-binding motif identified by Pleschke *et al*. (9). Conserved identical residues are in red. Asterisks denote residues mutated in this study. (**C**) Model of the BRCT1 domain based on the NMR structure (PDN accession code: 2D8M), highlighting the residues predicted to form phosphate-binding pocket. **(D)** Left, purified recombinant His-XRCC1^161–406^ and His-XRCC1^161–406 RK^ proteins, fractionated by SDS-PAGE and stained with coomassie brilliant blue. Right, cartoon of the *in vitro* PAR-binding assay. Proteins were adsorbed to microwell dishes and mock-ribosylated (‘-NAD^+^’) or ribosylated (‘+NAD^+^’) by PARP1 in absence or presence of NAD^+^ as indicated. Bound proteins were then incubated with recombinant wild type His-XRCC1^161–406^ or mutant His-XRCC1^161–406 RK^, and bound XRCC1 detected with anti-His tag antibodies colourmetrically (*A*_450_) using HRP-conjugated secondary antibody.

To further examine the importance of PAR binding for XRCC1 function we first addressed the location of the PAR-binding site. The most evolutionary conserved and functionally important region of XRCC1 is the central BRCT1 domain that mediates binding to PAR (see Figure 1A) (28). PAR-binding by the BRCT1 domain was initially ascribed to a degenerate motif of hydrophobic/basic amino acids that is present in many PAR binding proteins (Figure 1B, dotted red box) (9). However, a different putative PAR-binding motif in BRCT1 was recently reported, comprised of the phosphate-binding pocket common to several other BRCT domains (Figure 1B, solid red boxes and Figure 1C) (16). Within this pocket, Ser328, Arg335 and Lys369 are all predicted to contribute to phosphate binding, based on the structure of other phosphate-binding BRCT domains of this type. Consequently, for subsequent analysis *in vitro*, we expressed and purified both a wild-type histidine-tagged fragment of human XRCC1 spanning the conserved BRCT1 domain (denoted His-XRCC1^161–406^) and a mutant derivative in which both Arg335 and Lys369 were mutated to Ala (denoted His-XRCC1^161–406 RK^) (Figure 1D, left). We employed both mutations because mutation of R335 alone failed to have any measurable impact on XRCC1 function (data not shown).

**Figure 2.**
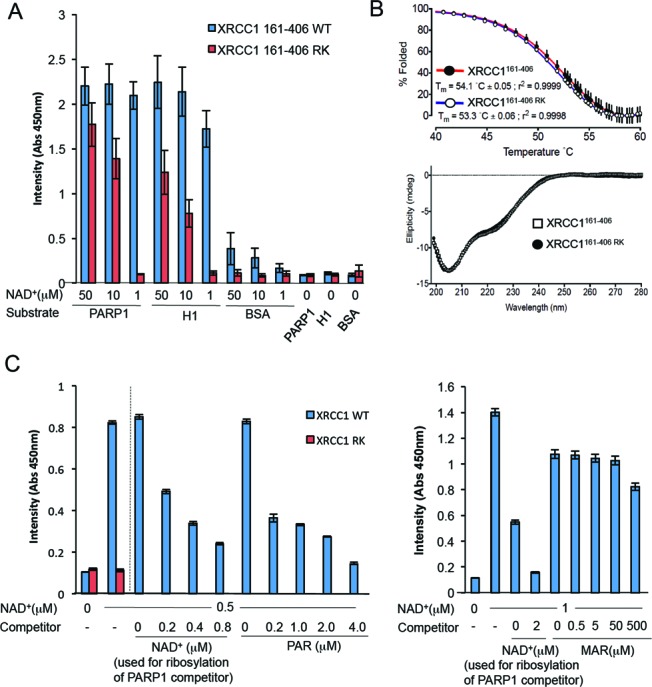
The XRCC1 BRCT1 phosphate-binding pocket binds PAR, *in vitro*. (**A**) Binding of His-XRCC1^161–406^ and His-XRCC1^161–406 RK^ to the indicated mock-ribosylated (-NAD^+^) or ribosylated (1–50 μM NAD^+^) proteins was measured as indicated in Figure 1D. Data are the mean (±1 SD) of at least three experiments. (**B**) Top, thermal stability of recombinant His-XRCC1^161–406^ and His-XRCC1^161–406 RK^. 2 μM XRCC1 protein was assayed in the presence of SYPRO Orange and unfolding temperatures determined as described in materials and methods. Data are the mean (±1SD) of four independent measurements. Bottom, circular dichroism of His-XRCC1^161–406^ and His-XRCC1^161–406 RK^. Data are the average of 10 sequential scans, with the spectrum from sample buffer alone subtracted. (**C**) Binding of His-XRCC1^161–406^ and His-XRCC1^161–406 RK^ to calf thymus histone mock-ribosylated in the absence of NAD+ (‘0’) or ribosylated in the presence of either 0.5 μM NAD^+^ (left panel) or 1 μM NAD^+^ (right panel). Where indicated, XRCC1 binding was measured in the presence of 43 nM PARP1 competitor that was first autoribosylated in the presence of 0, 0.2, 0.4, 0.8 or 2 μM NAD^+^, as indicated. Alternatively, His-XRCC1^161–406^ and His-XRCC1^161–406 RK^ binding was measured the presence of the indicated concentration of either poly (ADP-ribose) (‘PAR’, left) or mono (ADP-ribose) (‘MAR’, right) competitor. PAR/MAR competitor concentrations are total ADP-ribose units (μM) present as PAR (2–300 subunit lengths) or MAR. Data are the mean (±1SD) of at least three experiments.

Next, to confirm PAR binding by the BRCT1 phosphate-binding pocket we adsorbed PARP1, histone H1, or BSA to microwell plates, mock-ribosylated or ribosylated these proteins with PARP1 in the absence or presence of NAD^+^, respectively, and compared their binding to His-XRCC1^161–406^ and His-XRCC1^161–406 RK^, *in vitro* (Figure 1D, right). Wild-type His-XRCC1^161–406^ bound both to adsorbed PARP1 and histone H1, if these proteins were first ribosylated in the presence of 1–50 μM NAD^+^, and was fully bound even at the lowest concentration of NAD^+^ employed (1 μM) (Figure 2A, blue bars). In contrast, relatively little binding was observed to BSA, irrespective of whether or not it was first incubated with PARP1 and NAD^+^, consistent with this protein being a poor substrate for PARP1. More importantly, His-XRCC1^161–406 RK^ bound ribosylated PARP1 and histone H1 to a much lesser extent, and not at all at the lowest concentration (1 μM) of NAD^+^ employed (Figure 2A, red bars). This did not reflect a non-specific impact of the mutations on folding of the BRCT1 domain, because His-XRCC1^161–406^ and His-XRCC1^161–406 RK^ exhibited similar thermal stabilities and circular dichroism spectra (Figure 2B). Importantly, His-XRCC1^161–406^ bound specifically to PAR in these experiments, because it was suppressed by a 8-fold molar excess of ADP-ribose competitor if present as polymer (PAR), but was not suppressed even at 500-fold molar excess if present as ADP-ribose monomer (MAR) (Figure 2C). These data confirm that the phosphate-binding pocket of the XRCC1 BRCT1 domain promotes binding to PAR *in vitro*, particularly at low levels of poly (ADP-ribosylation).

**Figure 3.**
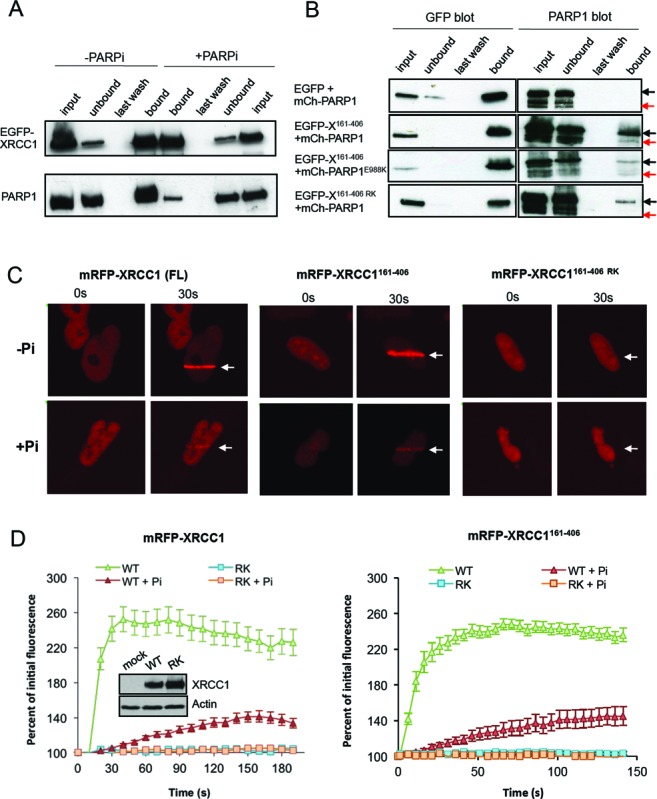
The XRCC1 BRCT1 phosphate-binding pocket mediates PAR-dependent interaction with PARP1 and recruitment at sites of UVA laser induced damage. (**A**) EGFP-XRCC1 was affinity purified from cell extract from U2OS^GFP-XRCC1^ cells using GFP-Trap beads. Aliquots of the column input, unbound, last wash, and eluate samples were fractionated by SDS-PAGE and immunoblotted with pS485/pT488 anti-XRCC1 polyclonal antibody or anti-PARP1 antibody. Where indicated (‘+PARPi’), PARP inhibitor (500 nM Ku-58948) was included in the cell lysis buffer and was present in the cell culture medium for 1 h at 37°C prior to lysis. (**B**) U2OS cells were transiently co-transfected with expression vector encoding either EGFP, EGFP-XRCC1^161–406^, or EGFP-XRCC1^161–406 RK^ and with expression vector encoding either mCherry-PARP1 or mCherry-PARP1^E988K^. EGFP-XRCC1 was recovered from whole cell extract and aliquots of column input, unbound, last wash and eluate (bound material) fractionated by SDS-PAGE and immunoblotted with anti-GFP or anti-PARP1 antibody. The position of mCherry-PARP1 and endogenous PARP1 are indicated by black and red arrows, respectively. (**C**) *XRCC1*-mutant EM9 cells were transiently transfected with pmRFP-XRCC1, pmRFP-XRCC1^RK^, pmRFP-XRCC1^161–406^, or pmRFP-XRCC1^161–406 RK^ and treated with UVA laser. mRFP fluorescence was measured at the indicated times (seco) following microirradiation in the presence or absence of 500nM PARP inhibitor (Ku-58948). Representative images are shown. (**D**) Left, quantitation of the mRFP-XRCC1 fluorescence proteins at sites of 405 nm UVA laser-induced DNA damage in the above experiments. Inset, pmRFP-XRCC1 and pmRFP-XRCC1^RK^ expression levels in the transfected cells, as measured by immunoblotting with pS485/pT488 anti-XRCC1 polyclonal antibody. Right quantitation of the mRFP-XRCC1^161^–^406^ fluorescence at sites of 351 nm UVA laser damage. Data is expressed as change in mean fluorescence in ten or more cells per construct ± SEM.

**Figure 4.**
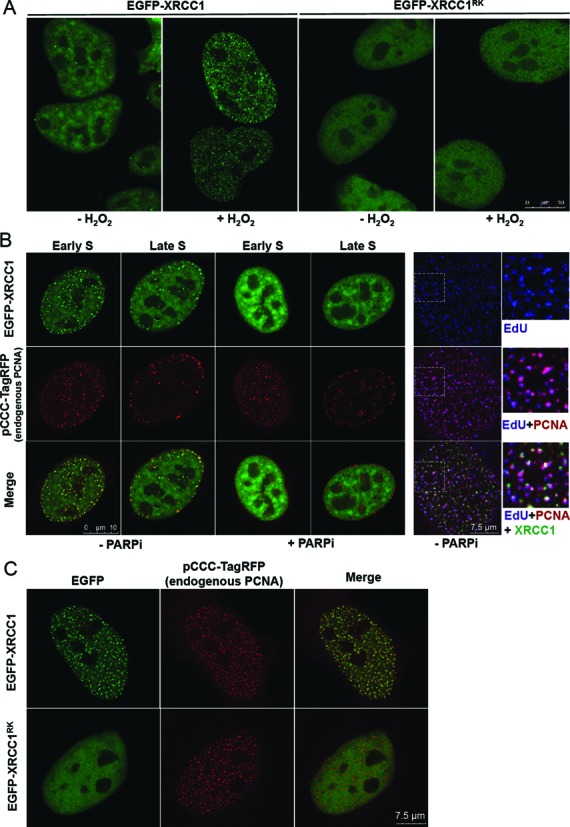
The XRCC1 BRCT1 phosphate-binding pocket is important for XRCC1 accumulation at sites of H_2_O_2_-induced damage and for colocalization with PCNA. (**A**) U2OS cells were transfected with pEGFP-XRCC1 or pEGFP-XRCC1^RK^, mock-treated or treated with 10 mM H_2_O_2_ for 10 min, and after 15 min recovery in drug-free medium fixed and analysed by fluorescence microscopy. (**B**) Left, U2OS^GFP-XRCC1^ cells were transfected with pCCC-TagRFP to detect endogenous PCNA in the presence or absence of the PARP inhibitor olaparib (100 nM) and analysed as above 24 h later. Right, cells were transfected as above and additionally pulse labelled with EdU (blue) to identify sites of DNA replication. Dotted square denotes the area expanded on the right. (**C**) U2OS cells were co-transfected with either pEGFP-XRCC1^WT^ or pEGFP-XRCC1^RK^ and pCCC-TagRFP plasmid to detect endogenous PCNA. Representative images are shown.

Next, we examined whether PAR binding by the phosphate-binding pocket is physiologically relevant, by comparing wild type and mutant XRCC1 for interaction with cellular PARP1. As expected, full length EGFP-XRCC1 co-precipitated endogenous PARP1 from stably transfected U2OS cells (U2OS^GFP-XRCC1^ cells; see 'Materials and Methods' section) in a manner that was inhibited by PARP inhibitor (Figure 3A). Similarly, truncated EGFP-XRCC1^161–406^ spanning the BRCT1 domain co-precipitated mCherry-PARP1 in transient co-transfection experiments, but co-precipitated mutant mCherry-PARP1^E988K^ lacking polymerase activity (29,30) to a much lesser extent (Figure 3B). More importantly, EGFP-XRCC1^161–406 RK^ was also less able to pull-down wild type mCherry-PARP1, confirming that the phosphate-binding pocket promotes interaction with PARP1 (Figure 3B). Consistent with these data, mRFP-XRCC1^161–406^ rapidly accumulated at sites of UVA laser damage at a rate similar to full-length mRFP-XRCC1 and in a manner that was greatly inhibited by PARP inhibitor (500 nM Ku58948), suggesting that the region spanning the BRCT1 domain is sufficient for XRCC1 accumulation at sites of cellular PAR synthesis (Figure 3C and D). Note that we confirmed previously that this concentration of Ku58948 greatly reduces or ablates PAR synthesis in UVA laser tracks (31). In contrast, neither full-length mRFP-XRCC1^RK^ nor mRFP-XRCC1^161–406 RK^ accumulated at sites of UVA laser damage (Figure 3C and D). Similarly, full-length EGFP-XRCC1^RK^ failed to accumulate in sub-nuclear foci at sites of H_2_O_2_-induced oxidative stress, confirming that the phosphate-binding pocket is also required for accumulation of EGFP-XRCC1 at this more physiologically relevant source of SSBs (Figure 4A).

XRCC1 has also been reported to colocalise with PCNA in replication foci in human cells, consistent with its proposed role during SSBR at sites of stalled or collapsed replication forks (1,32–35). However, whether XRCC1 accumulation at such sites is also regulated by PAR synthesis is not known. Indeed, the accumulation of EGFP-XRCC1 in sub-nuclear foci with endogenous PCNA, detected by expression of anti-PCNA antibody, was greatly reduced by PARP inhibitor in both early and late S phase cells (Figure 4B, left panels). We confirmed in these experiments that the sites of PCNA and EGFP-XRCC1 colocalisation were sites of DNA replication, by pulse labeling with EdU (Figure 4B, right panels). Importantly, EGFP-XRCC1 accumulation at sites of PCNA accumulation was greatly reduced or ablated by mutation of the phosphate-binding pocket, suggesting that PAR binding is also critical for the recruitment/accumulation of EGFP-XRCC1 at sites of DNA replication (Figure 4C).

**Figure 5.**
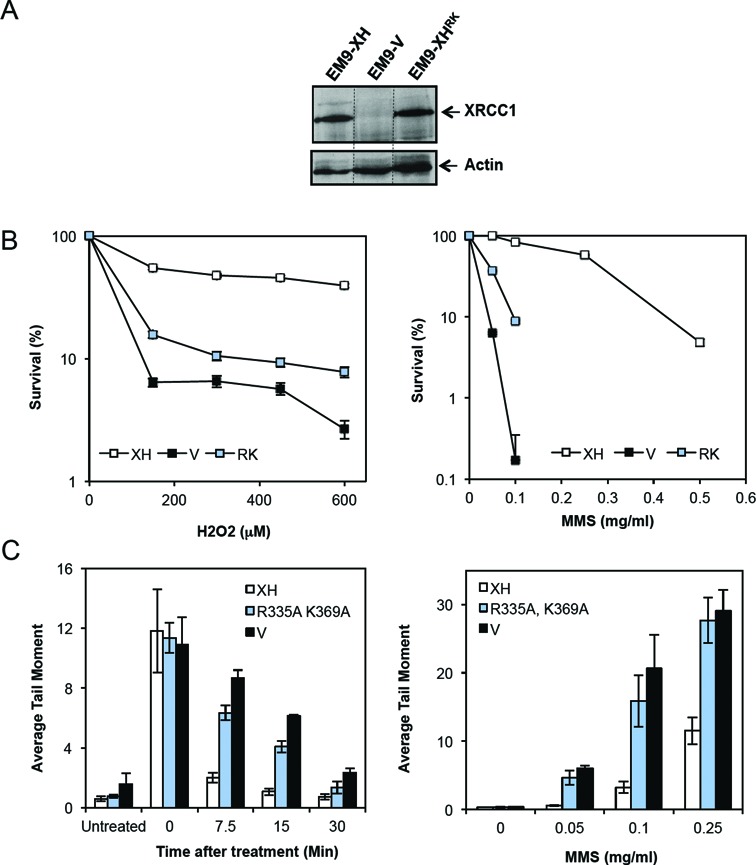
XRCC1-mediated acceleration of SSBR and cell survival requires the XRCC1 BRCT1 phosphate-binding pocket. (**A**) XRCC1 protein expression in *XRCC1-mutant* EM9 cells stably transfected with empty expression vector (EM9-V) or expression vector encoding either XRCC1-His (EM9-XH) or XRCC1-His^RK^ (EM9-XH^RK^). Cell extracts were fractionated by SDS-PAGE and immunoblotted with anti-XRCC1 Mab (33–2–5) and anti-Actin antibodies. (**B**) Clonogenic survival of *XRCC1*-mutant EM9 cells stably transfected with empty expression vector (EM9-V) or expression vector encoding either XRCC1-His (EM9-XH) or XRCC1-His^RK^ (EM9-XH^RK^). Cells were treated with the indicated concentrations of H_2_O_2_ (left) or MMS (right) for 15 min and then in drug free medium for 10–14 days to allow colony formation. Data are the mean (±SEM) of three independent experiments. Where not visible, error bars are smaller than the symbols. (**C**) Chromosomal SSBR rates were measured in the above EM9 cell lines in alkaline comet assays following treatment with 150 μM H_2_O_2_ for 20 min on ice, followed by recovery in drug-free medium for the indicated time at 37°C, or with the indicated concentration of MMS for 15 min at 37°C to measure the accumulation of SSB intermediates during BER. Data are the mean (±SEM) of three independent experiments.

Finally, to address the importance of the phosphate-binding pocket for XRCC1 function, we employed derivatives of *XRCC1*-mutant EM9 cells stably transfected with either empty vector (EM9-V) or with expression vector encoding either full-length human XRCC1-His (EM9-XH) or XRCC1-His^RK^ (EM9-XH^RK^) (Figure 5A). In contrast to XRCC1-His, XRCC1-His^RK^ was unable to promote cell survival in *XRCC1*-mutant EM9 cells much more than empty vector, following H_2_O_2_ or MMS treatment (Figure 5B). This was also true in experiments in which we measured rates of chromosomal SSBR using alkaline comet assays, in which XRCC1-His^RK^ again failed to correct the slow rate of DNA strand break repair observed in EM9 cells (Figure 5C).

Collectively, these data demonstrate that the XRCC1 phosphate-binding pocket binds PAR *in vitro* and in cells, promotes XRCC1 accumulation at sites of DNA damage, and is required for XRCC1 cellular function.

## DISCUSSION

The synthesis of poly (ADP-ribose) (PAR) by PARP1 can accelerate SSBR, but the molecular mechanism by which PAR achieves this is unclear (17). One likely role is promoting recruitment of the SSBR scaffold protein, XRCC1 (11–15), although this idea has proved controversial (18–22). To further address this possibility we have clarified the mechanism of PAR binding by XRCC1 and addressed its importance for SSBR and cell survival. PAR binding was initially ascribed to a degenerate motif present at the C-terminus of the central BRCT1 domain in XRCC1, comprised of an alternating series of basic/hydrophobic residues and present in numerous other PAR binding proteins (9). Interestingly, this motif in XRCC1 harbours a common polymorphism at amino acid 399 (arginine/glutamine), which in some epidemiological studies has been implicated in altered predisposition to cancer. However, in cellular assays this polymorphism does not impact measurably on XRCC1 function, suggesting that it does not influence PAR binding (36). Moreover, replacement of five of the basic residues characteristic of this degenerate motif with alanine also fails to impact on XRCC1 function, suggesting that the degenerate motif is not, by itself at least, important for PAR binding (unpublished observations).

Recently, PAR binding by XRCC1 was assigned to a different region of the BRCT1 domain; the highly conserved phosphate binding pocket in (16). In agreement with Li *et al*., we found that the phosphate-binding pocket interacts directly with PAR. However, in contrast to Li *et al*., we did not detect binding to mono(ADP-ribose) (MAR) by this motif. Indeed, our competition assays indicate that binding by this motif is highly selective for PAR. We found that the phosphate-binding pocket confers on XRCC1 the ability to bind PAR at low concentrations of polymer, as indicated by its greater impact on PAR binding by XRCC1 at low concentrations of NAD^+^, *in vitro*. This might be an advantage at low levels of SSBs such as those arising endogenously in cells, in which PAR polymer might be present at a low concentration and distributed at only a small number of sites across the genome. However, XRCC1 harbouring a mutated phosphate-binding pocket still bound PAR at high concentrations of polymer, albeit to a lesser extent than wild type XRCC1. This may reflect incomplete ablation of PAR binding by the R335A/K369A mutation or, alternatively, weak PAR binding conferred by the degenerate PAR binding motif described above. Nevertheless, mutation of the phosphate-binding pocket greatly reduced mRFP-XRCC1 recruitment at sites of UVA laser-induced damage, and also EGFP-XRCC1 at sites of DNA damage induced by H_2_O_2_, suggesting that this pocket is critical for accumulation of EGFP-XRCC1 at cellular sites of DNA strand breakage. Interestingly, the impact of mutating the phosphate-binding pocket on XRCC1 accumulation at sites of UVA laser induced damage was greater than incubation with PARP inhibitor. This might reflect incomplete inhibition of PAR synthesis by inhibitor or, alternatively, a low level of protein ribosylation generated prior to incubation with PARP inhibitor.

Mutation of the phosphate-binding pocket also greatly reduced XRCC1 accumulation at sites of PCNA accumulation, suggesting that PAR synthesis also promotes XRCC1 accumulation at sites of damaged replication forks. The latter is consistent with our model for replication-coupled SSBR, in which XRCC1 promotes repair of SSBs either ahead of an approaching fork or after replication fork collapse (35,37). It is also consistent with a role for PARP1 in regulating fork progression in the presence of DNA strand breaks (38–41). However, it is important to note that we have so far only observed XRCC1 accumulation at sites of ongoing DNA replication in cells co-expressing RFP-PCNA or anti-PCNA antibody (data not shown). Consequently, we suggest that both approaches perturb normal PCNA function to some extent, thereby generating SSBs and/or other sources of replication stress that trigger PARP1 activation.

Finally, XRCC1 harbouring a mutated phosphate-binding pocket was unable to restore rapid rates of chromosomal SSBR to *XRCC1*-mutant EM9 cells, following treatment with either H_2_O_2_ or MMS, and only slightly increased cellular resistance to these genotoxins. This work thus highlights the importance of the PAR-binding motif for XRCC1 functionality, both at oxidative breaks induced by H_2_O_2_ and following MMS-induced DNA alkylation. The latter is particularly intriguing, because MMS-induced SSBs arise as intermediates of DNA base excision repair (BER), suggesting that PAR is important for XRCC1 function during BER. Whereas several reports have suggested that PARP1 is required during BER following DNA alkylation (42,43), others have reported that it is dispensable (44–46). To reconcile this discrepancy, we previously suggested that PARP1 may be required to detect SSBs arising during BER only if the SSB intermediate becomes uncoupled from the canonical pathway, and/or during replication-coupled SSBR (35,37). However, in the current work, the PAR-binding pocket appeared to be required to accelerate most if not all XRCC1-dependent BER events, as measured by alkaline comet assays following MMS treatment. The extent to which PAR synthesis promotes BER events thus warrants further investigation.

In summary, we confirm that PAR binding is mediated by the phosphate-binding pocket of the XRCC1 BRCT1 domain, and show that the PAR-binding pocket promotes XRCC1 accumulation at DNA damage globally across the genome and at sites of DNA replication stress. We also show that the phosphate-binding pocket is required for acceleration of SSBR by XRCC1, and for XRCC1-dependent cell survival, supporting the hypothesis that poly (ADP-ribose) synthesis is important for XRCC1 recruitment and function.
